# Eicosanoids in Nonalcoholic Fatty Liver Disease (NAFLD) Progression. Do Serum Eicosanoids Profile Correspond with Liver Eicosanoids Content during NAFLD Development and Progression?

**DOI:** 10.3390/molecules25092026

**Published:** 2020-04-27

**Authors:** Dominika Maciejewska, Arleta Drozd, Karolina Skonieczna-Żydecka, Marta Skórka-Majewicz, Karolina Dec, Karolina Jakubczyk, Anna Pilutin, Ewa Stachowska

**Affiliations:** 1 Department of Human Nutrition and Metabolomics, Pomeranian Medical University in Szczecin, 70-204 Szczecin, Poland; arleta.drozd@gmail.com (A.D.); karzyd@sci.pum.edu.pl (K.S.-Ż.); marta_skorka@o2.pl (M.S.-M.); karolina_dec@wp.pl (K.D.); jakubczyk.kar@gmail.com (K.J.); ewast@pum.edu.pl (E.S.); 2 Department of Histology and Embryology, Pomeranian Medical University in Szczecin, 70-204 Szczecin, Poland; anna.pilutin@pum.edu.pl

**Keywords:** eicosanoids, NAFLD, NASH, 9-HODE, 13-HODE, biomarkers

## Abstract

Nonalcoholic fatty liver disease (NAFLD) is becoming a major public health problem worldwide. The study aimed to evaluate the concentration of eicosanoids in serum and liver tissue during steatosis progression and to assess whether eicosanoid change scores may predict liver tissue remodeling. Thirty six eight-week-old male Sprague Dawley rats were enrolled and sacrificed at different stages of NAFLD. Eicosanoid concentrations, namely lipoxin A_4_, hydroxyeicosatetraenoic acids (HETE), hydroxyloctadecadienoic acids (HODE), protectin DX, Maresine1, leucotriene B_4_, prostaglandin E_2_, and resolvin D_1_ measurement in serum and liver tissue with Agilent Technologies 1260 liquid chromatography were evaluated. For the liver and serum concentrations of 9-HODE and 13-HODE, the correlations were found to be strong and positive (r > 0.7, *p* < 0.05). Along with NAFLD progression, HODE concentration significantly increased, and change scores were more abundant in the liver. The moderate positive correlation between liver and serum (r = 0.52, *p* < 0.05) was also observed for resolvin E1. The eicosanoid concentration decreased during NAFLD progression, but mostly in serum. There were significant correlations between HETE concentrations in liver and serum, but their associations were relatively low and changes the most in liver tissue. Eicosanoids profile, predominantly 9-HODE and 13-HODE, may serve as a potential biomarker for NAFLD development.

## 1. Introduction

Nonalcoholic fatty liver disease (NAFLD) is becoming a major public health problem worldwide, affecting more than 25% of adults in the world population [1]. The NAFLD prevalence increases along with body fat content, which results in a 90% incidence of NAFLD in the obese [2,3]. The NAFLD phenotype ranges from simple steatosis to nonalcoholic steatohepatitis (NASH) and fibrosis [4]. The most important goal for NAFLD regression is the implementation of a healthy lifestyle. It was estimated that 10% body mass reduction significantly improved the stage of liver steatosis, inflammation, and fibrosis [5]. However, patients often do not follow lifestyle changes and the majority of long-term interventions do not give relative positive results [6].

NAFLD diagnosis is based on a histological pattern of steatosis type accompanied with no other chronic liver diseases [7]. However, liver biopsy also has several limitations, such as representing only a small area (approximately 1/50,000) of the organ. Therefore, there is a great need to develop non-invasive methods useful in NAFLD assessment [8]. There are several serum biomarkers essential for NAFLD evaluation. The most important are: Fatty liver index (FLI), Hepatic steatosis index (HSI), SteatoTest, NAFL screening score, Cytokeratin-18 (CK18), and Fibroblast growth factor 21 (FGF21) [6]. NAFD progression has been found to be linked with increased oxidative stress. Therefore, lipid peroxidation products have been considered as a diagnostic marker for NAFLD, especially for NASH progression. Lipid peroxidation products are synthesized via free radical-mediated direct oxidation and enzymatic pathways with lipoxygenases, cyclooxygenases, and cytochrome P450. Arachidonic acid (AA), linoleic acid (LA), eicosapentaenic acid (EPA), or docosahexaenoci acid (DHA) are able to produce multiple isomers of oxidized fatty acids (eicosanoids), all of which may activate the immune system [9,10]. Increased oxidation stress and lipid peroxidation have been demonstrated to induce the production of inflammatory cytokines, thus activating Kupffer cells and hepatic stellate cells, and consequently, hepatocyte apoptosis and liver fibrosis [11]. Emerging concepts in NASH assessment utilize oxidized fatty acids. The oxNASH model includes the ratio of linoleic acid and its most common oxidized form, 13-hydroksyloctadecadieenoic acid (13-HODE). When combined with age, body mass index (BMI), and asparagine aminotransferase (AST), the LA:13-HODE ratio was reported to be helpful in the prediction of NAFLD development [12].

The aim of our study was to evaluate the concentration of oxidized fatty acids in serum and liver tissue during steatosis progression with NASH and fibrosis development.

Because LA and AA metabolites are frequently described as oxidation markers in liver diseases, we attempted to assess whether serum oxidized fatty acids/eicosanoids correspond with liver eicosanoids content during NAFLD development and progression.

## 2. Materials and Methods

### 2.1. Animals

The study was carried out in 36 8-week-old male Sprague Dawley rats. The animals were separated into plastic cages (3 rats per cage). The rats were kept in rooms with heating and temperature control, in 12 h light/darkness cycles, and they had *ad libitum* access to food and water. In order to induce hepatic steatosis [13], the rats (n = 36, 6 groups, 6 rats/group) received a high-fat and high-cholesterol diet as previously described by Xu et al. [14]. The animals were sacrificed after 2, 4, 8, 12, 16, and 20 weeks of high-fat diet exposure. The rats were sacrificed by intraperitoneally injection of ketamine solution and bled via cardiac puncture. All procedures involving animals were carried out with international standards of animal care guidelines. The study was approved by the Local Ethical Committee on Animal Testing in Poznan, Poland (approval No 76/2016, 16.12.2016). No rat died in either group during the whole experimental period.

### 2.2. Histological Evaluation

For histological examination, the livers were immediately taken, fixed in 4% buffered formalin solution, embedded in paraffin, and cut into 4-μm sections. For the morphological analysis, serial sections of livers were stained with hematoxylin-eosin. Hepatic fibrosis was assessed by Mallory trichrome methods (Bio-Optica, Italy). Ten light microscopic fields were viewed on each section and scored for the severity of hepatic steatosis and fibrosis. For hepatic steatosis, the following criteria were used: grade 0—no fat; grade 1—steatosis occupying less than 33% of the hepatic parenchyma; grade 2—steatosis occupying less than 34–66% of the hepatic parenchyma; grade 3—more than 66% of the hepatic parenchyma. The following criteria were used to evaluate the staging of hepatic fibrosis: 0—none; 1—mild, zone 3, perisinusoidal; 2—moderate, zone 3, presinusoidal; 3—portal/periportal; 4—bridging fibrosis [15]. For inflammatory cell infiltration, the following criteria were used: grade 0—none; 1–1/2field, 2–3/4 field; 3—more than 4 foci/field [14]. NAFLD development is presented in Table 1. These results have been previously described by our team [13].

### 2.3. Sample Preparation

Lipoxin A_4_ (LX A_4_), 5-hydroxyeicosatetraenoic acid (5-HETE), 12-hydroxyeicosatetraenoic acid (12-HETE), 15-hydroxyeicosatetraenoic acid (15-HETE), 16-hydroxyeicosatetraenoic acid (16-HETE), 9-hydroxyloctadecadienoic acid (9-HODE), 13-hydroxyloctadecadienoic acid (13-HODE), 16-hydroxyeicosapentaenoic acid (16-HEPE), 17-hydroxydocosahexaenoic acid (17-HDHA), Protectin DX, Maresine1, Leucotriene B_4_, Prostaglandin B_2_, and resolvin D_1_ were extracted from the 0.5 mL of plasma by using a solid-phase extraction RP-18 SPE columns (Agilent Technologies, UK).

### 2.4. Instrumentation

The HPLC separations were performed on an Agilent Technologies 1260 liquid chromatography, consisting of model G1379B degasser, a model G1312B bin pump, a model G1316A column oven, and a model G1315CDAD VL+. Samples were injected using a model G1329B. An Agilent ChemStation software (Agilent Technologies, Cheadle, UK) was used for instrument control and data acquisition and analysis. The separation was completed on a Thermo Scientific Hypersil BDS C18 column 100 × 4.6 mm × 2.4 μm (cat no. 28102–154630). The temperature of column oven was set at 21 °C.

### 2.5. HPLC Operating Parameters

A gradient method was used, where the mobile phase was composed of a mixture of solvent A (methanol/water/acetic acid, 50/50/0.1, v/v/v) and B (methanol/water/acetic acid, 100/0/0.1, v/v/v). The percent content of buffer B in the mobile phase was 30% at 0.0 min to 2.00 min of separation, increased linearly to 80% at 33 min. It was 98% between 33.1 and 37.5 min and 30% between 40.3 and 45 min. The flow rate was 1.0 mL/min. The sample injection volume was 60 uL. The DAD detector monitored peaks by adsorption at 235 nm for 16-HEPE, 17-HDHA, 9-HODE, 13-HODE, 5-HETE, 12-HETE, and 15-HETE, at 280 nm for PGB2 (Prostaglandin B_2_, internal standard), resolvin E1, Protectin DX, Maresine1, Leucotriene B4 at 210 nm for Prostaglandin E2, 16-HETE, and at 302 nm for Lipoxin A4 and resolvin D1. Absorbance spectra of peaks were analyzed to confirm the identification of analytes. The quantitation was based on peak areas with internal standard calibration. Quantitative analysis was made by using ChemStation Software (Agilent Technologies, Cheadle, UK).

### 2.6. Statistical Analysis

The statistical analysis was performed using the “R 3.0.2” computer software. The normality of continuous variables distribution by means of Shapiro-Wilk test was evaluated. Consequently, parametric tests were used. Data are presented as means and standard deviation (SD). Student test (paired t-test) was used to analyze the differences between the groups. In order to estimate the correlation between outcomes of interest, the Pearson’s correlation test was used. The values of *p* < 0.05 were considered as statistically significant.

## 3. Results

During HFD exposure, thus NAFLD progression, significant alterations were observed, particularly in fatty acid metabolites. Table 2 and Table 3 present eicosanoids concentrations in serum and liver tissue by groups with different stages of NAFLD.

During the next step of the study, we estimated whether the metabolites measured in liver and serum correlate with one another. Table 4 presents the correlation coefficients between particular metabolites measured in different origin samplings. The moderate, positive correlation for resolvin E_1_ was found; strong correlations between serum and liver concentrations of 13-HODE and 9-HODE were depicted.

**Figure 1:**
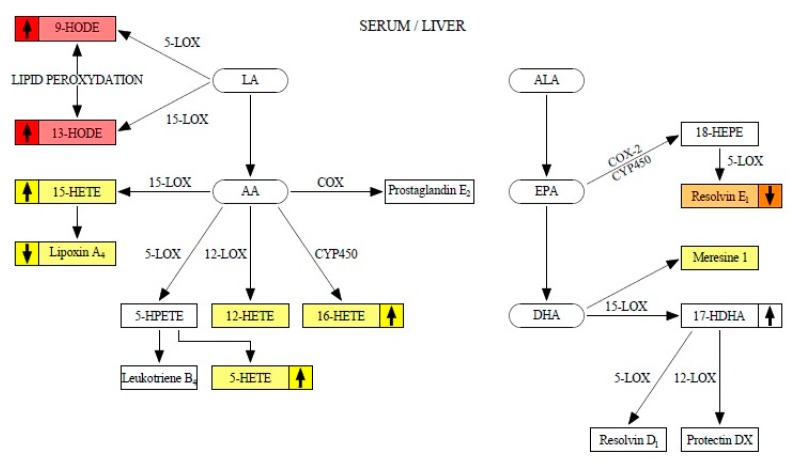
Eicosanoids metabolism.

Figure 1 shows the metabolism of eicosanoids analyzed during the study. The metabolites with weak liver-serum correlations are marked in yellow. The moderate correlation is marked in orange. Strong liver-serum correlations are marked in red. The up/down arrows indicate a tendency to increase/decrease particular eicosanoid concentration during steatosis progression.

## 4. Discussion

Chronic inflammation plays a significant role in the etiopathogenesis of cardiovascular entities, cancers, metabolic syndrome, and NAFLD [16]. Common drugs involved in the eicosanoids metabolizing pathways are effective in the treatment of metabolic disorders, suggesting a link between these molecules’ metabolism and inflammation [16]. The inflammation during NAFLD development affects not only the liver tissue, but the whole body. Therefore, eicosanoids concentration in serum represents the systemic inflammation [17].

The recent data demonstrated that the development of NAFLD was correlated with an increase in serum eicosanoids. Loomba et al. conducted a study where they compared the eicosanoid profile in patients with biopsy-proven simply steatosis and NASH. They found that NASH patients had significantly higher concentration of 15-HETE and PGE2 [18]. Feldstein et al. examined 49 patients with simply steatosis, NASH, and healthy individuals. The increase in peak areas of 13-HODE were observed in patients diagnosed with NASH compared to patients with simple steatosis and patients with normal liver biopsy. 9-HODE and 13-HODE also positively correlated with disease severity [19]. Puri et al. were able to show that patients with NASH had significantly higher concentration of hepatic 5-HETE, 15-HETE, 8-HETE, and 11-HETE compared with controls and those with simple steatosis, however they did not measure eicosanoid concentration in serum/plasma [19].

In the previous study of our group, we found that eicosanoid profile may serve as a good predictor for liver tissue remodeling. The pilot study in patients with early stages of NAFLD showed that concentration of 9-HODE in plasma differed between the first and second stage of liver steatosis. Moreover, after a successfully ended six-month dietary intervention, patients in both groups significantly reduced the levels of all of measured eicosanoids [13]. Another research demonstrated that in NAFLD, with individuals who reduced their body mass by more than 7% of total body weight, a significant improvement in the stage of steatosis, waist circumference, fatty liver index, triglycerides, and cholesterol took place. A reduction of body mass by more than 7% but not less than 7% of total body weight resulted in a significant improvement in steatosis stage, waist circumference, fatty liver index, and levels of triglycerides, cholesterol, as well as 5-lipoxygenase products: 5-oxo-ETE and LX A_4_. Liver steatosis and insulin resistance were significantly associated only with two eicosanoids-5-HETE and 5-oxo-ETE (also products of 5-lipooxygenase) [20].

The present study aimed answer the question: do serum eicosanoids profile correspond with liver eicosanoids content during NAFLD development and progression? The strongest positive correlation between liver and serum concentrations were found for 9- and 13-HODE (r > 0.7, *p* < 0.05). These two eicosanoids were also found to be significantly elevated during NAFLD progression, both in serum and the liver (Table 2 and Table 3). Based on these results, we could speculate that serum is less sensitive to steatosis than the liver. As far as 9- and 13-HODE concentrations in serum are concerned, these parameters increased significantly during the fibrosis and inflammation development in the liver (after 16, 20 weeks of HFD). Different results were noticed in liver tissue. The elevation was more evident between the groups. The study of ours confirms previous reports that 9- and 13-HODE are good predictors of NASH and fibrosis development [12]. Raszeja-Wyszomirska et al. described that 9-HODE and 13-HODE were significantly increased in NAFLD patients and patients with alcoholic liver disease (ALD). Their results demonstrated that the highest concentration of HODE were in ALD patients [21]. HODE play an important role in increase induction of vasodilatation [22], inhibition of the intracellular calcium [23], suppression of proliferation, and apoptosis induction. Moreover, Zhang et al. revealed that 13-HODE induced ER stress, perturbed lipid metabolism, and enhanced protein levels of fatty acid synthase, which progress the inflammation in the liver [24].

The moderate correlation (r = 0.52, *p* <0.05) between liver and serum resolvin E1 concentration was observed. The resolvin potentially has anti-inflammatory properties [25], including inhibition of polymorphonuclear leukocytes (PMN) infiltration [26] regulation of phagocytosis-induced apoptosis, promotion of macrophage efferocytosis [27], and reduction of pro-inflammatory cytokines [28]. Due to the fact that most analyses focus on pro-inflammatory factors as lipid biomarkers of NAFLD progression, the data on the impact of resolvin E_1_ in NAFLD evaluation are still insufficient. However, a recent study demonstrated that omega-3 derivatives, including resolvin E_1_, exerts protective actions in the liver by preventing necrotic and inflammatory injury in the organ [29]. Resolvin E_1_ and D_1_ decrease the progression of liver injury in mouse model [30]. Our results showed that concentration of the eicosanoid decreased during NAFLD progression, but mostly in serum. Significant differences in liver tissue were noticed between a group exposed to HFD for 2 weeks (with no steatosis) and for 20 weeks (a group with inflammation and fibrosis).

The literature data provides the evidence that HETE compounds are potential markers of metabolic disease. 5-HETE concentration was found to be increased in obesity, metabolic syndrome, and NAFLD [31]. 12-HETE affected chemokine profile and exaggerated the inflammation within fatty liver [32], while 15-HETE was demonstrated to characterize the progression from normal to simple steatosis to NASH [33]. Some authors describe 15-HETE as an anti-inflammatory eicosanoid due to its conversion to lipoxin A_4_ (Figure 1) by endogenous macrophage-derived 5-LOX activity [34]. However, our study showed that during NAFLD progression, lipoxin A_4_ decreased in both serum and liver, and the conversion was reduced. All HETEs have highly inflammatory properties and their concentration increased during NAFLD progression, especially in NASH development [35]. Our study revealed that HETE (5-, 12-, 15-, and 16-HETE) in serum significantly correlate with HETE concentration in liver tissue, but the strengths of the correlations were low. We could demonstrate a significant increase between groups, but mostly in the liver tissue. Similar results were observed by Puri et al. [19].

## 5. Conclusions

Eicosanoids profile, predominantly 9-HODE and 13-HODE, could potentially serve as biomarkers for NAFLD development, reflecting the disease progression in both serum and the liver. Apart from anti-inflammatory mediators that are able to predict liver tissue alterations, resolvin E_1_ was found to significantly differ between NAFLD stages, stating this molecule as the next biomarker of NAFLD development. The levels of 5-, 12-, 15-, and 16-HETE were also linked to NAFLD phenotype, but the association was found to be weak.

## 6. Limitations

The study consists of novel observations, but it also has limitations. We do not measure gene expression related to eicosanoids metabolism. The profile of eicosanoids in our study is not wide enough, especially as anti-inflammatory ones are concerned. Thus, the possibility of another eicosanoids linkage with NAFLD marker does exist.

## Figures and Tables

**Table 1:** Scores of hepatic steatosis, inflammation, and hepatic fibrosis.

Group	Week	N (n)	Histological Grades of Steatosis and Inflammation				Fibrosis Stage (N)				
			Number of Evaluated Histological Fields (Percentage of Grade of Steatosis/Inflammation)								
			0	1	2	3	0	1	2	3	4
**STEATOSIS**	2	6 (60)	360 (100)	0 (0)	0 (0)	0 (0)	6	0	0	0	0
	4	6 (60)	45 (75) ^a^	15 (25) ^a^	0 (0)	0 (0)	6	0	0	0	0
	8	6 (60)	0 (0)^a,b^	47(78,3) ^a,b^	13(21,7) ^a,b^	0 (0)	6	0	0	0	0
	12	6 (60)	0 (0) ^a,b^	0 (0) ^bc^	38(63,33) ^a,b,c^	22(36,67) ^a,b,c^	1	4	1	0	0
	16	6 (60)	0 (0) ^a,b^	0 (0) ^b,c^	26(43,33) ^a,b,c^	34(56,67) ^a,b,c,d^	1	3	2	0	0
	20	6 (60)	0 (0) ^a,b^	0 (0) ^b,c^	15(25) ^b,d,e^	45(75) ^a,b,c,d,e^	1	1	4	0	0
**INFLAMMATION**	2–20	36(360)	360 (100)	0 (0)	0 (0)	0 (0)					
	2	6 (60)	360 (100)	0 (0)	0 (0)	0 (0)					
	4	6 (60)	48 (80)	12 (20)	0 (0)	0 (0)					
	8	6 (60)	41 (68,33) ^a^	18 (30) ^a^	1 (1,67)	0 (0)					
	12	6 (60)	38 (63,33) ^a,b^	18 (30) ^a^	4 (6,67) ^a,b^	0 (0)					
	16	6 (60)	28 (46,66) ^a,b,c^	25 (41,67) ^a,b^	7 (11,67) ^a,b,c^	0 (0)					
	20	6 (60)	26 (43,34) ^a,b,c,d^	26 (43,33) ^a,b^	8 (11,33) ^a,b,c^	0 (0)					

Data of steatosis are expressed as counts and percentages in parentheses. N–number of animals (n)—number of evaluated histological fields. ^a^ *p* < 0.05 vs. NAFLD 2 weeks, ^b^ *p* < 0.05 vs. NAFLD 4 weeks, ^c^ *p* < 0.05 vs. NAFLD 8 weeks, ^d^ *p* < 0.05 vs. NAFLD 12 weeks, ^e^ *p* < 0.05 vs. NAFLD 16 weeks.

**Table 2:** Serum eicosanoids concentration during Nonalcoholic Fatty Liver Disease (NAFLD) progression.

Eicosanoids in Serum [ug/mL]	2 Weeks		4 Weeks		8 Weeks		12 Weeks		16 Weeks		20 Weeks	
	Mean	SD	Mean	SD	Mean	SD	Mean	SD	Mean	SD	Mean	SD
**Resolvin E_1_**	**34.29 ^8,12,16,20^**	**12.95**	**18.90 ^12,16,20^**	**13.71**	**12.18 ^16,20^**	**7.61**	**11.01 ^16,20^**	**7.65**	**7.17 ^20^**	**4.43**	**1.73**	**0.81**
Prostaglandin E_2_	2.89	0.62	3.53	1.56	2.83	1.62	4.57	2.82	3.48	2.18	2.83	1.53
Resolvin D_1_	0.01	0.01	0.00	0.01	0.00	0.01	0.00	0.01	0.01	0.01	0.01	0.01
**LX A_4_**	**0.14 ^8,12,16,20^**	**0.10**	**0.13 ^8,12,16,20^**	**0.09**	**0.03**	**0.03**	**0.02**	**0.03**	**0.03**	**0.05**	**0.04**	**0.03**
Protectin DX	0.41	0.10	0.29	0.10	0.42	0.19	0.36	0.13	0.40	0.17	0.43	0.09
Maresine1	0.18	0.07	0.16	0.05	0.20	0.21	0.12	0.01	0.22	0.09	0.11	0.02
**Leucotriene B_4_**	**0.19 ^20^**	**0.13**	**0.21 ^20^**	**0.07**	**0.11**	**0.08**	**0.13**	**0.04**	**0.11**	**0.09**	**0.08**	**0.01**
18-HEPE	0.31	0.10	0.21	0.05	0.30	0.27	0.31	0.28	0.22	0.10	0.26	0.04
**16-HETE**	**1.50**	**1.38**	**1.22**	**0.80**	**1.14 ^12,16,20^**	**1.68**	**1.92**	**1.16**	**1.88**	**1.45**	**1.98**	**0.45**
**13-HODE**	**0.45 ^8,12,16,20^**	**0.18**	**0.55 ^8,12,16,20^**	**0.17**	**0.62 ^20^**	**0.22**	**0.64 ^20^**	**0.27**	**0.69 ^20^**	**0.11**	**0.81**	**0.02**
**9-HODE**	**0.32 ^16,20^**	**0.17**	**0.38 ^16,20^**	**0.04**	**0.37 ^16,20^**	**0.09**	**0.42**	**0.22**	**0.45**	**0.11**	**0.49**	**0.03**
15-HETE	2.12	1.45	2.95	1.41	1.73	0.34	1.97	1.50	1.97	0.52	1.90	0.33
17-HDHA	0.56	0.07	0.71	0.14	0.84	0.36	0.64	0.32	0.75	0.36	0.72	0.08
**12-HETE**	**39.22 ^12^**	**71.28**	**42.24**	**10.31**	**21.13**	**7.19**	**9.32**	**2.27**	**16.12**	**6.66**	**29.85**	**9.72**
5-HETE	0.25	0.06	0.27	0.08	0.30	0.18	0.28	0.21	0.28	0.08	0.35	0.04

**^4^**^,**8**,**12**,**16**,**20**^ Significant differences between particular group (*p* < 0.05).

**Table 3:** Liver eicosanoids concentration during NAFLD progression.

Eicosanoids in Liver [ug/mL]	2 Weeks		4 Weeks		8 Weeks		12 Weeks		16 Weeks		20 Weeks	
	Mean	SD	Mean	SD	Mean	SD	Mean	SD	Mean	SD	Mean	SD
**Resolvin E_1_**	**4.4 ^8,20^**	**2.9**	**2.7**	**1.5**	**2.5**	**1.8**	**2.8**	**1.7**	**2.6**	**1.5**	**1.8**	**1.4**
Prostaglandin E_2_	3.6	2.1	5.6	5.8	3.6	5.2	3.6	2.9	3.0	2.9	3.0	4.0
Resolvin D_1_	0.6	0.6	0.6	0.2	0.6	0.2	0.7	0.3	0.6	0.6	0.7	0.7
**LX A_4_**	**0.6 ^20^**	**0.2**	**0.6 ^20^**	**0.3**	**0.5 ^16^**	**0.3**	**0.5**	**0.5**	**0.5**	**0.9**	**0.3**	**0.4**
Protectin DX	0.5	0.2	0.6	0.2	0.8	0.3	0.4	0.3	0.9	0.6	0.5	0.4
**Maresine 1**	**1.3 ^12,16,20^**	1.1	1.9	1.2	1.7	0.6	1.9	0.8	2.9	1.6	2.8	1.8
Leucotriene B_4_	0.9	0.3	1.8	0.7	1.6	0.6	1.1	0.4	1.5	0.9	1.0	0.8
**18-HEPE**	**3.3 ^12^**	2.7	4.3	2.8	4.5	1.4	3.4	2.8	4.5	2.2	3.9	3.4
**16-HETE**	**3.5 ^16,20^**	**3.1**	**5.8**	**2.3**	**5.1**	**2.0**	**5.3**	**2.1**	**6.6**	**2.4**	**8.0**	**7.7**
**13-HODE**	**102.7 ^12,16,20^**	**82.8**	**114.3 ^12,16,20^**	**30.1**	**131.8 ^12,16,20^**	**43.5**	**149.4 ^16,20^**	**84.7**	**191.5**	**118.7**	**185.9**	**178.2**
**9-HODE**	**90.2 ^8,12,16,20^**	**71.1**	**104.1 ^8,12,16,20^**	**26.6**	**110.3 ^12,16,20^**	**33.5**	**128.1 ^16,20^**	**77.5**	**171.2**	**105.9**	**168.3**	**165.6**
**15-HETE**	**62.7 ^4,8,12,16,20^**	**44.3**	**138.3 ^8,12,16,20^**	**38.4**	**135.1 ^12,16,20^**	**56.3**	**109.0 ^16,20^**	**59.9**	**154.8 ^20^**	**87.5**	**158.5**	**111.5**
**17-HDHA**	**5.8 ^16,20^**	**3.7**	**10.6 ^20^**	**4.9**	**7.3 ^20^**	**4.0**	**10.6 ^20^**	**10.5**	**15.5**	**12.2**	**36.9**	**24.8**
**12-HETE**	**39.1**	**32.6**	**59.8**	**52.9**	**23.3 ^12,16^**	**14.4**	**24.5**	**18.1**	**39.2**	**21.1**	**40.1**	**21.8**
**5-HETE**	**1.1 ^4,8,12,16,20^**	**0.4**	**1.8 ^8,12,16,20^**	**0.6**	**2.9 ^16,20^**	**1.2**	**2.2 ^16,20^**	**2.0**	**4.7**	**1.8**	**4.1**	**3.7**

**^4^**^,**8**,**12**,**16**,**20**^ Significant differences between particular group (*p* < 0.05).

**Table 4:** Correlation of eicosanoids concentration between serum and liver tissue.

Eicosanoids [ug/mL]	r	*p*
**Resolvin E_1_**	**0.52**	***p*** **< 0.01**
Prostaglandin E_2_	0.21	NS
Resolvin D_1_	0.19	NS
**LX A_4_**	**0.32**	***p*** **< 0.05**
DiHDHA	0.03	NS
**Maresine 1**	**0.31**	***p*** **< 0.01**
Leucotriene B_4_	0.07	NS
18-HEPE	0.31	NS
**16-HETE**	**0.38**	***p*** **< 0.01**
**13-HODE**	**0.72**	***p*** **< 0.01**
**9-HODE**	**0.71**	***p*** **< 0.01**
**15-HETE**	**0.43**	***p*** **< 0.01**
17-HDHA	0.52	***p* < 0.01**
**12-HETE**	**0.39**	***p*** **< 0.01**
**5-HETE**	**0.35**	***p*** **< 0.05**
